# Decreased levels of serum glutathione peroxidase 3 are associated with papillary serous ovarian cancer and disease progression

**DOI:** 10.1186/1757-2215-4-18

**Published:** 2011-10-22

**Authors:** Deep Agnani, Olga Camacho-Vanegas, Catalina Camacho, Shashi Lele, Kunle Odunsi, Samantha Cohen, Peter Dottino, John A Martignetti

**Affiliations:** 1 Department of Genetics and Genomic Sciences, Mount Sinai School of Medicine, New York, NY 10029, USA; 2 Department of Gynecologic Oncology, Roswell Park Cancer Institute, Buffalo, New York 14263, USA; 3 Department of Obstetrics, Gynecology, and Reproductive Science, Mount Sinai School of Medicine, New York, NY 10029, USA; 4 Department of Pediatrics, Mount Sinai School of Medicine, New York, NY 10029, USA; 5 Department of Oncological Sciences, Mount Sinai School of Medicine, New York, NY 10029, USA

**Keywords:** Ovarian cancer, Papillary serous carcinoma, Glutathione peroxidase 3, GPX3

## Abstract

**Background:**

Glutathione peroxidase 3 (GPX3) is a selenocysteine-containing antioxidant enzyme that reacts with hydrogen peroxide and soluble fatty acid hydroperoxides, thereby helping to maintain redox balance within cells. Serum levels of GPX3 have been found to be reduced in various cancers including prostrate, thyroid, colorectal, breast and gastric cancers. Intriguingly, GPX3 has been reported to be upregulated in clear cell ovarian cancer tissues and thus may have implications in chemotherapeutic resistance. Since clear cell and serous subtypes of ovarian cancer represent two distinct disease entities, the aim of this study was to determine GPX3 levels in serous ovarian cancer patients and establish its potential as a biomarker for detection and/or surveillance of papillary serous ovarian cancer, the most frequent form of ovarian tumors in women.

**Patients and Methods:**

Serum was obtained from 66 patients (median age: 62 years, range: 22-89) prior to surgery and 65 controls with a comparable age-range (median age: 53 years, range: 25-83). ELISA was used to determine the levels of serum GPX3. The Mann Whitney *U *test was performed to determine statistical significance between the levels of serum GPX3 in patients and controls.

**Results:**

Serum levels of GPX3 were found to be significantly lower in patients than controls (p = 1 × 10^-2^). Furthermore, this was found to be dependent on the stage of disease. While levels in early stage (I/II) patients showed no significant difference when compared to controls, there was a significant reduction in late stage (III/IV, p = 9 × 10^-4^) and recurrent (p = 1 × 10^-2^) patients. There was a statistically significant reduction in levels of GPX3 between early and late stage (p = 5 × 10^-4^) as well as early and recurrent (p = 1 × 10^-2^) patients. Comparison of women and controls stratified to include only women at or above 50 years of age shows that the same trends were maintained and the differences became more statistically significant.

**Conclusions:**

Serum GPX3 levels are decreased in women with papillary serous ovarian cancer in a stage-dependent manner and also decreased in women with disease recurrence. Whether this decrease represents a general feature in response to the disease or a link to the progression of the cancer is unknown. Understanding this relationship may have clinical and therapeutic consequences for women with papillary serous adenocarcinoma.

## Background

Epithelial ovarian cancer (EOC) is the most lethal of all gynecologic cancers and the fifth most frequent cause of female cancer deaths [1]. It is estimated that over 21,000 new cases and 13,000 deaths will be attributed to the disease in 2011 alone [1]. Although 5-year survival rates have increased over the past several decades to approximately 40%, overall mortality rates remain relatively constant [1] largely because most women present late in disease course with widespread intra-abdominal metastasis. Five-year relative survival rates drop from > 90% for disease diagnosed at an early stage to < 30% for disease diagnosed in later stages [2].

Currently, no serum biomarker has been FDA approved for the early detection of ovarian cancer whereas CA125 and the recently approved human epididymis protein 4 (HE4) are being utilized to monitor disease progress [2,3]. While several biomarkers/panels of biomarkers with reported higher sensitivities and specificities than CA125 are being investigated, none of these have improved upon the low efficacy of the measurement of CA125 levels in distinguishing ovarian cancer patients from controls during the asymptomatic stages of the disease [4-10]. Recently, the OVA1™ test representing a biomarker panel and analysis based on menopausal status has received FDA approval for preoperative evaluation of ovarian cancer risk in women with an ovarian mass [11]. Interestingly, levels of three of the five biomarkers, apolipoprotein, prealbumin and transferrin, decrease in women with malignancy. This suggests that the search for biomarkers should expand beyond tumor-specific overexpressed proteins.

Tumor growth results in oxidative stress, accompanied by an increase in reactive oxygen species (ROS). ROS serve as secondary messenger molecules and may result in increased cellular proliferation, an increase in genetic mutations and overall genetic instability, increased cellular invasion and angiogenesis [12]. ROS are also known to stimulate pathways that may lead to development of drug resistance in cancer cells [13]. Higher levels of ROS are, however, toxic to cells and cancer treatments often employ strategies to increase ROS production [14]. Increases in the levels of ROS also lead to the increase in transcription of antioxidant enzymes including catalase, superoxide dismutase, glutathione-*S*-transferase, and glutathione peroxidase [12-16]. Thus the differential expression of antioxidant enzymes in cancer could serve as biomarkers of disease initiation and/or progression.

One antioxidant enzyme whose expression in serum/plasma has been correlated with various cancers is glutathione peroxidase 3 (GPX3) [17]. A number of studies have shown GPX3 activity to be downregulated in patients with breast, gastric and colorectal cancers [18]. GPX3 was also found to be uniformly downregulated in all grades of endometrial adenocarcinoma, both in rats as well as humans, irrespective of tumor grade [19]. Furthermore, sera of glioblastoma patients appear to have lower levels of GPX3 when compared to controls [20]. On a genetic level, downregulation of GPX3 via hypermethylation of its promoter has been described in human esophageal squamous cell carcinoma tissue [21] and primary prostrate cancer samples and cell lines [22,23].

Intriguingly, previous studies have shown that compared to control tissues GPX3 expression is higher in clear cell epithelial ovarian carcinoma tissue [24-26]. Clear cell cancers account for approximately 5% of all ovarian cancers. The most common histology of ovarian cancer is papillary serous (> 60%) and the other histologies include endometrioid (~25%) and mucinous (~5%) cancers. A proteomic analysis of women with stage IV papillary serous carcinoma who had been previously treated with surgery and chemotherapy also revealed the presence of GPX3 in their ascites fluid [27]. It is important to note that serum levels of GPX3 were not examined in either the clear cell or late-stage previously treated studies. Given that papillary serous epithelial ovarian cancer represents the majority of ovarian tumors and that no previous studies have examined serum GPX3 levels in women with this histology of ovarian cancer, we therefore hypothesized that GPX3 may represent a novel biomarker for this disease.

## Materials and methods

### Serum sample collection

A total of 66 serum samples from patients and 65 serum samples from controls with a comparable age-range were examined. Serum samples were obtained from three different sources: Twenty-eight (20/22 early, and 8/31 late stage) patient samples were from the Roswell Park Cancer Institute, Buffalo, NY, USA; twenty (20/65) control serum samples were commercially obtained from Bioserve Biotechnologies, Ltd. (Beltsville, MD, USA). All other samples, along with the relevant clinical data, were obtained from blood samples collected at the Mount Sinai School of Medicine (MSSM). Studies were approved by the respective medical ethics committees.

At MSSM, blood samples were collected in BD Vacutainer SST™ Plus Blood Collection Tubes (BD Biosciences, USA). Samples were spun down at 2600 rpm for 10 minutes at 4°C in Eppendorf 5810R centrifuge (Eppendorf, USA) to separate serum. Samples were then stored at -130°C until ELISA assay was performed.

### ELISA assay

Commercially available ELISA kits for measuring concentrations of GPX3, manufactured by Adipogen™ and supplied by ENZO Lifesciences, USA were obtained. All samples were diluted at 1:250 ratio in buffer provided in the kit. Assays were performed as per manufacturers' instructions, using the provided standard curve reagents. Controls and samples were run in duplicate to assure consistency. Intra-sample variability was less than 10%.

### Statistical analyses

A two-sided Mann-Whitney *U *test was performed in MATLAB R2009B (The Mathworks, Inc., Natick, MA, USA) to compare GPX3 levels between groups. A p-value of less than 0.05 was considered to be statistically significant. All box-plots were performed using Excel.

## Results

### Patients

Serum samples from 66 patients with pathology-confirmed papillary serous ovarian cancer and 65 healthy controls were examined. Patient characteristics are shown in Table 1. The median age for the patients was 62 years (range: 22-89) while that of the controls was 53 (range: 25-83). Incorporated into the analysis were clinical factors including age, stage of disease and histological grade. As shown in Table 1, we selected for a higher number of early stage samples beyond the usual expected frequency of these cases in an unbiased population to specifically determine if there was a significant change in the levels of GPX3 in these samples.

**Table 1:** Sample demographics and clinicopathologic characteristics

Characteristic	Number of Patients (%)	Number of Controls (%)
**Ethnicity**		
***Caucasian***	28 (42.4)	27 (41.6)
***African-American***	2 (3)	1 (1.5)
***Other***	6 (9.1)	1 (1.5)
***Unknown***	30 (45.5)	36 (55.4)

**Age (Years)**		
***Median***	62 (range: 22-89)	53* (range: 25-83)

**Ovarian Cancer Stage**		
***Early (Stage 1/2)***	22 (33)	
***Late (Stage 3/4)***	31 (47)	
***Recurrent***	13 (20)	

**Histological Grade**		
***Well differentiated (1)***	6 (9)	
***Moderately Differentiated (2)***	15 (23)	
***Poorly differentiated (3)***	39 (59)	
***Unknown***	6 (9)	

* n = 50; age of 15 controls had not been recorded. The values in brackets represent percentage to total.

### GPX3 serum levels are lower in patients when compared to controls

**Figure 1:**
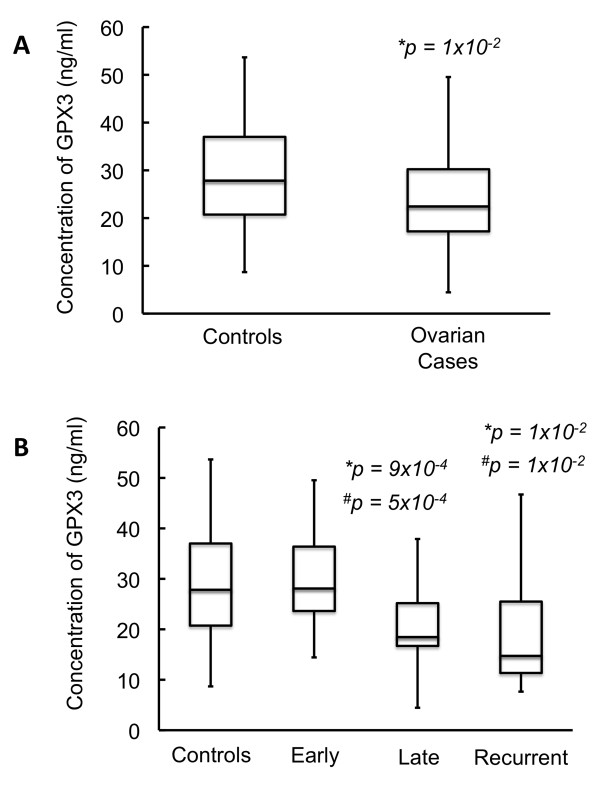
**Comparison of GPX3 levels of healthy female controls vs. women with serous ovarian cancer for women of all ages: Figure 1A shows a group-wise comparison of GPX3 in healthy female controls vs. women diagnosed with papillary serous ovarian cancer while Figure 1B shows a stage-wise comparison of GPX3 in healthy female controls vs. women diagnosed with papillary serous ovarian cancer**. Star (*) denotes statistically significant decrease in GPX3 expression when compared to controls. Hash (#) denotes statistically significant difference in GPX3 expression when compared to early stage samples. Women diagnosed with serous ovarian cancer show a statistically significant decrease in the levels of GPX3. A stage-wise examination shows that there is a significant decrease in GPX3 levels in late stage and recurrent cancer. There is also a significant difference in levels of GPX3 between patients with early and late stage/recurrent disease.

A Mann Whitney *U *test was performed comparing GPX3 concentrations between serum from all patients and controls. GPX3 concentrations were significantly lower in patients than controls (median value of 22.4 ng/ml in patients, compared to 27.8 ng/ml in controls, p = 1 × 10^-2^, Figure 1A). We next explored if GPX3 levels correlated with stage (Figure 1B). Women with late stage disease (median, 18.5 ng/ml; p = 9 × 10^-4^) and recurrence of their cancer (median, 14.7 ng/ml; p = 1 × 10^-2^) had significantly lower levels of GPX3 than controls. No difference was identified between women with early stage disease and controls (p = 0.6). In addition women with late stage disease (p = 5 × 10^-4^) and recurrence of their cancer (p = 1 × 10^-2^) had significantly lower levels of GPX3 than women with early stage disease. These results are summarized in Table 2.

**Figure 2:**
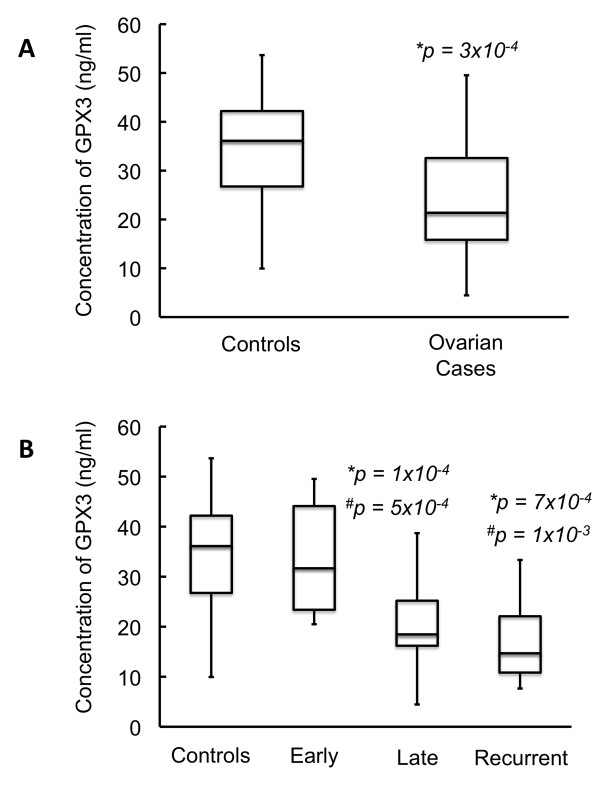
**Comparison of GPX3 levels of healthy female controls vs. women with serous ovarian cancer ≥ 50 years of age (average age of menopause): Figure 2A shows a group-wise comparison of GPX3 in healthy female controls vs. women diagnosed with papillary serous ovarian cancer while Figure 2B shows a stage-wise comparison of GPX3 in healthy female controls vs. women diagnosed with papillary serous ovarian cancer**. Star (*) denotes statistically significant decrease in GPX3 expression when compared to controls. Hash (#) denotes statistically significant difference in GPX3 expression when compared to early stage samples. Women diagnosed with serous ovarian cancer show a statistically significant decrease in the levels of GPX3. A stage-wise examination shows that there is a significant decrease in GPX3 levels in late stage and recurrent cancer. There is also a significant difference in levels of GPX3 between patients with early and late stage/recurrent disease.

**Table 2:** Summary of data from Figure 1A and 1B

Variable	Control	All Patients	Early	Late	Recurrent
**Number of samples (n)**	65	66	22	31	13

**GPX3 concentration (ng/ml)**					
***Median***	27.8	22.4	28.1	18.5	14.7
***Maximum***	53.7	49.5	49.5	44.6	48.9
***Minimum***	8.7	4.5	14.4	4.5	7.7

**Statistical Analysis: Mann Whitney *U *test (p-value)**					
***Vs. Controls***		**1 × 10^-2^**	0.6	**9 × 10^-4^**	**1 × 10^-2^**
***Vs. Early stage samples***				**5 × 10^-4^**	**1 × 10^-2^**

Comparison of all samples indicates that GPX3 levels significantly decrease in patients and are correlated with stage. p-values indicating statistically significant differences are shown in bold.

Since most ovarian cancer cases are diagnosed in postmenopausal women, we next compared the levels of GPX3 between controls and patients such that we included only women ≥ 50 years of age in each group. When stratified by age, GPX3 levels were even more significantly lower in all patients (21.4 ng/ml) when compared to controls (36.1 ng/ml; p = 3 × 10^-4^). In this age-delimited population, the differences were again even more significant in women with late stage disease (median, 18.5 ng/ml; p = 1 × 10^-4^) and recurrence (median, 14.7 ng/ml; p = 7 × 10^-4^). A statistically significant reduction in levels of GPX3 in patients diagnosed with late stage (p = 5 × 10^-4^) and recurrent disease (p = 1 × 10^-3^) when compared to those diagnosed with early stage disease was again present. These results are summarized in Table 3.

**Table 3:** Summary of data from Figure 2A and 2B

Variable	Control	All Patients	Early	Late	Recurrent
**Number of samples (n)**	30	56	16	29	11

**GPX3 concentration (ng/ml)**					
***Median***	36.1	21.4	31.7	18.5	14.7
***Maximum***	53.7	49.5	49.5	44.6	33.4
***Minimum***	9.9	4.5	20.5	4.5	7.7

**Statistical Analysis: Mann Whitney *U *test (p-value)**					
***Vs. Controls***		**3 × 10^-4^**	0.5	**1 × 10^-4^**	**7 × 10^-4^**
***Vs. Early stage samples***				**5 × 10^-4^**	**1 × 10^-3^**

Comparison of samples ≥ 50 years indicates that GPX3 levels significantly decrease in patients and are correlated with stage with an even greater statistical significance than that seen in Table 1. p-values indicating statistically significant differences are shown in bold.

No statistically significant correlations of GPX3 concentrations were identified with age, ethnicity or grade of disease (data not shown).

## Discussion

Using a candidate-based approach, and samples from 3 independent sources, we have identified that the serum protein GPX3, a selenocysteine-containing antioxidant enzyme, is decreased in women with serous ovarian cancer in a stage-dependent manner. In addition, we demonstrate that serum levels are also decreased in women with recurrent disease and the stage-dependent decreases are more pronounced when patients and controls are stratified to include only those women > 50 years of age. Thus, while a number of other studies have examined GPX3 levels in a broad array of cancer (Table 4), these studies provide the first analysis of this candidate biomarker in epithelial ovarian cancer, specifically, the serum of women with papillary serous ovarian cancer.

**Table 4:** GPX3 associations with cancer

Cancer Type	Overexpression/ Downregulation	RNA/ Protein	Cell/Tissue/ Serum/Plasma	Species	References
Esophageal Squamous Cell	Downregulation	mRNA, protein	Tumor tissue	Human	[31]
Gastric, Cervical, Thyroid, Head, Neck, Lung and Melanoma	Downregulation	mRNA, protein	Tumor tissue	Human	[32]
Thyroid	Downregulation	mRNA	Tumor tissue	Human	[33]
Ovarian Clear Cell	Upregulation	mRNA	Cell Lines	Human	[25]
Ovarian Clear Cell	Upregulation	mRNA, protein	Tumor tissue	Human	[24]
Ovarian Clear Cell	Upregulation	mRNA	Tumor tissue	Human	[26]
Ovarian Papillary Serous: Late Stage/previously treated	Presence	Protein	Ascites Fluid	Human	[27]
Glioblastoma	Downregulation	mRNA, protein	Tumor tissue, Serum	Human	[20]
Meningioma	Downregulation	mRNA	Tumor Tissue	Human	[34]
Lung	Downregulation	protein	Whole blood, Plasma (activity measurement)	Human	[35]
Endometrial adenocarcinoma	Downregulation	mRNA	Tumor Tissue	Human, Rat	[19]
Barrett's adenocarcinoma	Downregulation	mRNA	Tumor Tissue	Human	[36]
Prostate	Downregulation	mRNA	Tumor tissue	Human	[23]
Lung	Upregulation	protein	Blood serum	Mouse	[37]
Prostate cancer	Upregulation	protein	Blood serum	Human, Rat	[38]

Oncogenesis is associated with an increase in the intracellular levels of ROS, in turn resulting in an upregulation of antioxidant enzymes [12-16]. However, several studies conducted on tissue as well as blood/serum samples have shown that levels of the antioxidant enzyme GPX3 are decreased in a number of human cancers, including breast, gastric, prostrate and colorectal cancer; a seemingly contradictory effect [18-21,28,29]. A number of recent studies in clear cell ovarian cancer tissues conducted by others have identified a higher expression of GPX3 when compared to control cells and in other epithelial ovarian cancer histologies [24-26]. This not only suggests a potential anomaly but also could have therapeutic consequences since higher levels of GPX3 have been shown to confer chemotherapeutic resistance in cells [25]. The only other study performed in papillary serous cancer examined the ascites fluid of women with advanced stage disease after their treatment with surgery and chemotherapy and who were being treated for removal of an accumulation of ascites fluid [27]. Since serous ovarian cancer represents the most common epithelial ovarian cancer histology, we wanted to specifically examine the serum levels of this epithelial ovarian cancer subtype.

Our results demonstrate that serum GPX3 is downregulated in serous ovarian cancer. More importantly we identified a statistically significant difference in GPX3 levels between early and late stage/recurrent patients, suggesting that GPX3 may serve as a biomarker of disease progression. These differences reach greatest statistical significance when patients/controls are stratified to include only women above 50 years of age, the age at which most cases are diagnosed.

It is interesting to note that inspection of our MSSM cohort identified a patient for whom GPX3 levels seemed more indicative of disease status than CA125. Specifically, one of our 58 year old women with stage IIIC disease had a CA125 level of 32.3 U/ml (within normal limits) but a low GPX3 level (17.7 ng/ml). It will therefore be interesting in the future to evaluate if GPX3 could be coupled with CA125 or other candidate biomarkers to increase their sensitivity and specificity.

Under normal conditions, ROS play a role in signal transduction [12,13,16]. However, higher levels of intracellular ROS can lead to increased DNA mutations that have been associated with increased carcinogenesis [12,13].

Cellular studies indicate that GPX3 physiologically serves as a first line of defense reducing ROS to harmless species prior to their entry into the cell [29]. While our studies clearly define decreased serum GPX3 levels in women with ovarian cancer, we are not able to distinguish whether the decrease may represent a risk factor for the development of the cancer or simply represents a systemic response to the disease. If the decrease is a risk factor, could GPX3 be used as a screening tool or could increases in GPX3 reduce lifetime risk? Similarly, if the decrease represents a response to the disease, do patients with different GPX3 levels have different disease outcomes or health sequelae? For example, in a study on critically ill patients in an intensive care setting, decreased GPX3 levels were associated with a systemic inflammatory response syndrome (SIRS) [30]. Thus important future studies will be validating these results and in exploring the role of GPX3 in cancer initiation, progression and outcome.

In conclusion, this study demonstrates that serum GPX3 levels are reduced in papillary serous ovarian cancer patients when compared to controls and that, at least in one instance, decreased levels of GPX3 may provide additional diagnostic information beyond CA125.

## Abbreviations

CA125: Mucin 16, cell surface associated; GPX3: Glutathione peroxidase 3; HE4: Human epididymis protein 4; MSSM: Mount Sinai School of Medicine; ROS: Reactive oxygen species; SIRS: Systemic inflammatory response syndrome.

## Competing interests

The authors declare that they have no competing interests.

## Authors' contributions

DA conceptualized and designed the experiments, collected, assembled, analyzed and interpreted data, and drafted the manuscript. OC conceptualized and designed the experiments, and collected, assembled and analyzed data. CC designed and implemented experiments. SS recruited, collected and annotated specimens, and interpreted data. KO recruited, collected and annotated specimens, and interpreted data. SC collected and annotated specimens, and analyzed and interpreted data. PD conceptualized and designed the experiments, analyzed and interpreted data, and helped with the drafting of manuscript. JM conceptualized and designed the experiments, analyzed and interpreted data, and helped with the drafting of manuscript. All the authors in this manuscript have read and approved the final version.

## References

[B1] Jemal A Bray F Center MM Ferlay J Ward E Forman D Global Cancer statistics CA Cancer J Clin 2011 61 69 90 10.3322/caac.20107 21296855

[B2] Ovarian Cancer Home Page-National Cancer Institute http://www.cancer.gov/cancertopics/types/ovarian

[B3] Kim YM Whang DH Park J Kim SH Lee SW Park HA Ha M Choi KH Evaluation of the accuracy of serum human epididymis protein 4 in combination with CA125 for detecting ovarian cancer: a prospective case-control study in a Korean population Clin Chem Lab Med 2011 49 527 534 10.1515/CCLM.2011.085 21320028

[B4] Zhu CS Pinsky PF Cramer DW Ransohoff DF Hartge P Pfeiffer RM Urban N Mor G Bast RC Jr Moore LE Lokshin AE McIntosh MW Skates SJ Vitonis A Zhang Z Ward DC Symanowski JT Lomakin A Fung ET Sluss PM Scholler N Lu KH Marrangoni AM Patriotis C Srivastava S Buys SS Berg CD PLCO Project Team A Framework for evaluating biomarkers for early detection: validation of biomarker panels for ovarian cancer Cancer Prev Res (Phila) 2011 4 375 383 10.1158/1940-6207.CAPR-10-0193 PMC3057372 21372037

[B5] Cramer DW Bast RC Jr Berg CD Diamandis EP Godwin AK Hartge P Lokshin AE Lu KH McIntosh MW Mor G Patriotis C Pinsky PF Thornquist MD Scholler N Skates SJ Sluss PM Srivastava S Ward DC Zhang Z Zhu CS Urban N Ovarian cancer biomarker performance in prostate, lung, colorectal, and ovarian cancer screening trial specimens Cancer Prev Res (Phila) 2011 4 365 374 10.1158/1940-6207.CAPR-10-0195 PMC3085251 21372036

[B6] Mai PL Wentzensen N Greene MH Challenges related to developing serum-based biomarkers for early ovarian cancer detection Cancer Prev Res (Phila) 2011 4 303 306 10.1158/1940-6207.CAPR-11-0053 PMC3077065 21372029

[B7] Petricoin EF Ardekani AM Hitt BA Use of proteomic patterns in serum to identify ovarian cancer Lancet 2002 359 572 577 10.1016/S0140-6736(02)07746-2 11867112

[B8] Zhang Z Bast RC Jr Yu Y Li J Sokoll LJ Rai AJ Rosenzweig JM Cameron B Wang YY Meng XY Berchuck A Van Haaften-Day C Hacker NF de Bruijn HW van der Zee AG Jacobs IJ Fung ET Chan DW Three biomarkers identified from serum proteomic analysis for the detection of early stage ovarian cancer Cancer Res 2004 64 5882 5890 10.1158/0008-5472.CAN-04-0746 15313933

[B9] Gorelik E Landsittel DP Marrangoni AM Modugno F Velikokhatnaya L Winans MT Bigbee WL Herberman RB Lokshin AE Multiplexed immunobead-based cytokine profiling for early detection of ovarian cancer Cancer Epidemiol Biomarkers Prev 2005 14 981 987 10.1158/1055-9965.EPI-04-0404 15824174

[B10] Visintin I Feng Z Longton G Ward DC Alvero AB Lai Y Tenthorey J Leiser A Flores-Saaib R Yu H Azori M Rutherford T Schwartz PE Mor G Diagnostic markers for early detection of ovarian cancer Clin Cancer Res 2008 14 1065 1072 10.1158/1078-0432.CCR-07-1569 18258665

[B11] Zhang Z Chan DW The road from discovery to clinical diagnostics: lessons learned from the first FDA-cleared in vitro diagnostic multivariate index assay of proteomic biomarkers Cancer Epidemiol Biomarkers Prev 2010 19 2995 2999 10.1158/1055-9965.EPI-10-0580 20962299 PMC4836873

[B12] Azad MB Chen Y Gibson SB Regulation of autophagy by reactive oxygen species (ROS): Implications for cancer progression and treatment Antioxid Redox Signal 2009 11 777 790 10.1089/ars.2008.2270 18828708

[B13] Pelicano H Carney D Huang P ROS stress in cancer cells and therapeutic implications Drug Resist Updat 2004 7 97 110 10.1016/j.drup.2004.01.004 15158766

[B14] Harris AL Hypoxia-A key regulatory factor in tumor growth Nat Rev Cancer 2002 2 38 47 10.1038/nrc704 11902584

[B15] Tertil M Jozkowicz A Dulak J Oxidative stress in tumor angiogenesis-therapeutic targets Curr Pharm Des 2010 16 3877 3894 10.2174/138161210794454969 21158725

[B16] Avni R Cohen B Neeman M Hypoxic stress and cancer: imaging the axis of evil in tumor metastasis NMR Biomed 2011 in press 10.1002/nbm.1632 PMC3558740 21793071

[B17] Brigelius-Flohé R Kipp A Glutathione peroxidases in different stages of carcinogenesis Biochim Biophys Acta 2009 1790 1555 1568 19289149 10.1016/j.bbagen.2009.03.006

[B18] Pawłowicz Z Zachara BA Trafikowska U Maciag A Marchaluk E Nowicki A Blood selenium concentrations and glutathione peroxidase activities in patients with breast cancer and with advanced gastrointestinal cancer J Trace Elem Electrolytes Health Dis 1991 5 275 277 1822338

[B19] Falck E Karlsson S Carlsson J Helenius G Karlsson M Klinga-Levan K Loss of glutathione peroxidase 3 expression is correlated with epigenetic mechanisms in endometrial adenocarcinoma Cancer Cell Int 2010 10 46 54 10.1186/1475-2867-10-46 21106063 PMC3014921

[B20] Sreekanthreddy P Srinivasan H Kumar DM Nijaguna MB Sridevi S Vrinda M Arivazhagan A Balasubramaniam A Hegde AS Chandramouli BA Santosh V Rao MR Kondaiah P Somasundaram K Identification of potential serum biomarkers of glioblastoma: serum osteopontin levels correlate with poor prognosis Cancer Epidemiol Biomarkers Prev 2010 19 1409 1422 10.1158/1055-9965.EPI-09-1077 20530493

[B21] He Y Wang Y Li P Zhu S Wang J Zhang S Identification of GPX3 epigenetically silenced by CpG methylation in human esophageal squamous cell carcinoma Dig Dis Sci 2011 56 681 688 10.1007/s10620-010-1369-0 20725785

[B22] Lodygin D Epanchintsev A Menssen A Diebold J Hermeking H Functional epigenomics identifies genes frequently silenced in prostate cancer Cancer Res 2005 15 4218 4227 10.1158/0008-5472.CAN-04-4407 15899813

[B23] Yu YP Yu G Tseng G Cieply K Nelson J Defrances M Zarnegar R Michalopoulos G Luo JH Glutathione peroxidase 3, deleted or methylated in prostate cancer, suppresses prostate cancer growth and metastasis Cancer Res 2007 67 8043 8050 10.1158/0008-5472.CAN-07-0648 17804715

[B24] Lee HJ Do JH Bae S Yang S Zhang X Lee A Choi YJ Park DC Ahn WS Immunohistochemical evidence for the over-expression of glutathione peroxidase 3 in clear cell type ovarian adenocarcinoma Med Oncol 2010 in press 10.1007/s12032-010-9659-0 20730571

[B25] Saga Y Ohwada M Suzuki M Konno R Kigawa J Ueno S Mano H Glutathione peroxidase 3 is a candidate mechanism of anticancer drug resistance of ovarian clear cell adenocarcinoma Oncol Rep 2008 20 1299 1303 19020706

[B26] Hough CD Cho KR Zonderman AB Schwartz DR Morin PJ Coordinately up-regulated genes in ovarian cancer Cancer Res 2001 61 3869 3876 11358798

[B27] Kuk C Kulasingam V Gunawardana CG Smith CR Batruch I Diamandis EP Mining the Ovarian Cancer Ascites Proteome for Potential Ovarian Cancer Biomarkers Mol Cell Proteomics 2009 8 661 9 10.1074/mcp.M800313-MCP200 19047685 PMC2667349

[B28] Sarto C Frutiger S Cappellano F Sanchez JC Doro G Catanzaro F Hughes GJ Hochstrasser DF Mocarelli P Modified expression of plasma glutathione peroxidase and manganese superoxide dismutase in human renal cell carcinoma Electrophoresis 1999 20 3458 3466 10.1002/(SICI)1522-2683(19991101)20:17<3458::AID-ELPS3458>3.0.CO;2-5 10608715

[B29] Howie AF Walker SW Akesson B Arthur JR Beckett GJ Thyroidal extracellular glutathione peroxidase: a potential regulator of thyroid-hormone synthesis Biochem J 1995 308 713 717 8948423 10.1042/bj3080713 PMC1136783

[B30] Manzanares W Biestro A Galusso F Torre MH Mañay N Pittini G Facchin G Hardy G Serum selenium and glutathione peroxidase-3 activity: biomarkers of systemic inflammation in the critically ill? Intensive Care Med 2009 35 882 889 10.1007/s00134-008-1356-5 19034425

[B31] Ye He Y Wang Y Li P Zhu S Wang J Zhang S Identification of GPX3 epigenetically silenced by CpG methylation in human esophageal squamous cell carcinoma Dig Dis Sci 2011 56 681 8 10.1007/s10620-010-1369-0 20725785

[B32] Zhang X Yang JJ Kim YS Kim KY Ahn WS Yang S An 8-gene signature, including methylated and down-regulated glutathione peroxidase 3, of gastric cancer Int J Oncol 2010 36 405 14 20043075

[B33] Schmutzler C Mentrup B Schomburg L Hoang-Vu C Herzog V Köhrle J Selenoproteins of the thyroid gland: expression, localization and possible function of glutathione peroxidase 3 Biol Chem 2007 388 1053 1059 10.1515/BC.2007.122 17937619

[B34] Fevre-Montange M Champier J Durand A Wierinckx A Honnorat J Guyotat J Jouvet A Microarray gene expression profiling in meningiomas: differential expression according to grade or histopathological subtype Int J Oncol 2009 35 1395 4077 19885562 10.3892/ijo_00000457

[B35] Zachara BA Marchaluk-Wisniewska E Maciaq A Peplinski J Skokowski J Decreased selenium concentration and glutathione peroxidase activity in blood and increase of these parameters in malignant tissue of lung cancer patients Lung 1997 175 321 332 10.1007/PL00007578 9270989

[B36] Lee OJ Schneider-Stock R McChesney PA Kuester D Roessner A Vieth M Moskaluk CA El-Rifai W Hypermethylation and loss of expression of glutathione peroxidase-3 in Barrett's tumorigenesis Neoplasia 2005 7 854 61 10.1593/neo.05328 16229808 PMC1501938

[B37] Chatterji B Borlak J A 2-DE MALDI-TOF study to identify disease regulated serum proteins in lung cancer of c-myc transgenic mice Proteomics 2009 9 1044 1056 10.1002/pmic.200701135 19180532

[B38] Fan Y Murphy TB Byrne JC Brennan L Fitzpatrick JM Watson RWG Applying Random Forests To Identify Biomarker Panels in Serum 2D-DIGE Data for the Detection and Staging of Prostate Cancer J Proteome Res 2011 10 1361 1373 10.1021/pr1011069 21166384

